# University of Pennsylvania

**Published:** 1899-04

**Authors:** L. A. Nolan

**Affiliations:** Secretary


Secretary.

### VETERINARY MEDICAL SOCIETY, UNIVERSITY OF PENNSYLVANIA.

THE meeting was called to order March 17, 1899, at 8 P.M. Mr. Newcomer was appointed critic.

The programme of the evening consisted of an address by Dr. Alexander Glass on the "Different Breeds of Dogs." The members of the Society were accorded the privilege of asking any questions they deemed advisable. All were very well pleased with the interesting and instructive lecture.

Upon the adjournment of the meeting the members gathered in the assembly room, where a light lunch and smoker were participated in.

Among those present were Drs. J. W. Adams, Alexander Glass, B. F. Senseman, C. J. Marshall, and J. H. McNeall. The honorary president, Dr. Adams, entertained the members in an admirable manner. This affair is also enrolled in the archives of the Society as one of the important events of student life.

L. A. NOLAN,
Secretary.

